# TGF‐β transactivates EGFR and facilitates breast cancer migration and invasion through canonical Smad3 and ERK/Sp1 signaling pathways

**DOI:** 10.1002/1878-0261.12162

**Published:** 2018-01-24

**Authors:** Yuanyuan Zhao, Jing Ma, Yanling Fan, Zhiyong Wang, Ran Tian, Wei Ji, Fei Zhang, Ruifang Niu

**Affiliations:** ^1^ Public Laboratory National Clinical Research Center for Cancer Tianjin Medical University Cancer Institute and Hospital China; ^2^ Key Laboratory of Cancer Prevention and Therapy Tianjin China; ^3^ Tianjin's Clinical Research Center for Cancer China; ^4^ Key Laboratory of Breast Cancer Prevention and Therapy Ministry of Education Tianjin Medical University China

**Keywords:** breast cancer, EGF receptor, invasion, Smad3, Sp1, transforming growth factor‐β

## Abstract

Transforming growth factor‐beta (TGF‐β) functions as a potent proliferation inhibitor and apoptosis inducer in the early stages of breast cancer, yet promotes cancer aggressiveness in the advanced stages. The dual effect of TGF‐β on cancer development is known as TGF‐β paradox, and the remarkable functional conversion of TGF‐β is a pivotal and controversial phenomenon that has been widely investigated for decades. This phenomenon may be attributed to the cross talk between TGF‐β signaling and other pathways, including EGF receptor (EGFR) signaling during cancer progression. However, the underlying mechanism by which TGF‐β shifts its role from a tumor suppressor to a cancer promoter remains elusive. In this study, TGF‐β is positively correlated with EGFR expression in breast cancer tissues, and a functional linkage is observed between TGF‐β signaling and EGFR transactivation in breast cancer cell lines. TGF‐β promotes the migration and invasion abilities of breast cancer cells, along with the increase in EGFR expression. EGFR is also essential for TGF‐β‐induced enhancement of these abilities of breast cancer cells. Canonical Smad3 signaling and ERK/Sp1 signaling pathways mediate TGF‐β‐induced EGFR upregulation. Hence, our study provided insights into a novel mechanism by which TGF‐β supports breast cancer progression.

**Abbreviations**
ChIP: Chromatin Immunoprecipitation
EGFR: EGF receptor
FBS: Fetal bovine serum
IHC: immunohistochemistry
MTM: mithramycin
qRT‐PCR: quantitative RT‐PCR
TGF‐β: transforming growth factor‐beta

## 1 Introduction

Transforming growth factor‐beta (TGF‐β) is initially identified for its ability to induce cellular transformation in normal fibroblasts (de Larco and Todaro, 1978; van Zoelen *et al*., 1992). TGF‐β is also a critical regulator of development and homeostasis by inhibiting cell proliferation and inducing apoptosis in most cell types (Tian *et al*., 2011). In mice, the loss of TGF‐β gene shows more increased probability of tumorigenesis (Tang *et al*., 1998). Loss‐of‐function mutations or deletions in TGF‐β pathway genes, including TGF‐β receptors and SMAD genes, are frequently observed in different cancer types (Blaker *et al*., 2002; Massague *et al*., 2000). These results suggest that TGF‐β signaling inhibits the tumor development of normal epithelial cells. Conversely, an increased TGF‐β expression is found in many tumors in advanced stages. TGF‐β also promotes cancer cell invasion ability *in vitro* and metastatic ability *in vivo* (Bellomo *et al*., 2016; Deckers *et al*., 2006; Heldin *et al*., 2012). The blockage of TGF‐β signaling can significantly inhibit the invasive and metastatic properties of various cancer cells (Bedi *et al*., 2012). These findings demonstrate the promoter role of TGF‐β signaling during cancer progression. Thus, TGF‐β signaling elicits dual effects on cancer development (Inman, 2011). This phenomenon suggests that tumor cells can overcome the growth inhibitory effect of TGF‐β and retain their oncogenic responses to TGF‐β signaling during cancer progression. Nevertheless, the mechanism by which TGF‐β switches its role from a tumor suppressor to a cancer promoter remains undefined.

EGF is another well‐known potent regulator of cellular functions. Unlike those of TGF‐β, the biological actions of EGF are motogenic and mitogenic in most cell types (Wee *et al*., 2015). The binding of EGF to an EGF receptor (EGFR) can promote cell survival and proliferation (Cohen *et al*., 1998; Prenzel *et al*., 2001). In addition, EGFR signaling also favors cell differentiation, migration, and invasion *in vitro* (Abd El‐Rehim *et al*., 2004; Liu *et al*., 2017; da Rosa *et al*., 2014). In contrast to TGF‐β signaling, EGF/EGFR signaling predominantly functions as a promoter in tumorigenesis (Arteaga and Engelman, 2014). Therefore, the balance of EGF and TGF‐β signaling is necessary to maintain tissue homeostasis, and the disrupted balance of pro‐proliferative and antiproliferative signaling is associated with carcinogenesis. Dysregulated EGFR signaling has been observed in many cancer types, including breast cancer, colon cancer, and lung cancer (Matalkah *et al*., 2016; Nautiyal *et al*., 2012). EGFR overexpression has also been associated not only with cancer progression but also with poor prognosis of patients with cancer (Scaltriti and Baselga, 2006; Wieduwilt and Moasser, 2008). TGF‐β signaling activation has also been detected in tumors in late stages, and this process further aggravates cancer in many experimental models *in vitro* and *in vivo*. Considering its antiproliferative and pro‐apoptotic functions in early stages of carcinogenesis, we supposed that the activation of the EGFR pathway may antagonize TGF‐β‐induced antigrowth response in cancer cells, and these two pathways synergistically cooperate to increase cancer cell aggressiveness.

The signaling initiated by TGF‐β is different from that triggered by the EGF/EGFR pathway. However, the cross talk between TGF‐β and EGFR signaling in promoting cancer progression has been observed in several tumor types. TGF‐β and EGF can synergistically promote malignant phenotypes, such as epithelial‐to‐mesenchymal transition, which is a crucial process in cancer invasion and metastasis (Richter *et al*., 2011; Uttamsingh *et al*., 2008; Xu *et al*., 2010). Although TGF‐β alone can promote the invasiveness of cancer, the addition of EGF can enhance this effect (Buonato *et al*., 2015; Xiong *et al*., 2014). TGF‐β stimulation can also augment the oncogenic activities of EGF (Wendt *et al*., 2010). Thus, the cooperation between TGF‐β and EGF/EGFR signaling pathways is likely beneficial to cancer progression. Therefore, how these two signaling pathways collaborate during cancer aggravation should be investigated.

In this study, the expression of TGF‐β and EGFR in breast cancer tissues was investigated. Our results revealed a positive correlation between TGF‐β and EGFR expression. Using breast cancer cells as experimental models, we observed a functional association between TGF‐β signaling and EGFR transactivation in breast cancer cell lines. We demonstrated that TGF‐β treatment induced a significant increase in EGFR expression in breast cancer cells, and EGFR was essential for TGF‐β‐induced enhancement of the migration and invasion abilities of breast cancer cells. Canonical Smad3 signaling and ERK/Sp1 signaling pathways were also crucial for the TGF‐β‐induced upregulation of EGFR expression and the enhancement of migration and invasion abilities of breast cancer cells. Our study proposed a novel mechanism by which TGF‐β transactivates EGFR signaling and favors the migration and invasion of breast cancer cells.

## 2 Materials and methods

### 2.1 Reagents and drugs

The following reagents and drugs were used: RPMI 1640 medium and trypsin (Hyclone, Logan, UT, USA); FBS (Gibco, Carlsbad, CA, USA); TGF‐β (#100‐21; PeproTech, Rocky Hill, NJ, USA); PD98059 and SB431542 and erlotinib (MedChem Express, Monmouth Junction, NJ, USA); mithramycin (MTM; #6891; Sigma‐Aldrich, St. Louis, MO, USA); Trizol reagent/Lipofectamine RNAmix/Lipofectamine 2000 (Invitrogen, Carlsbad, CA, USA); EDTA‐free protease inhibitor (Roche, Indianapolis, IN, USA); Transwell inserts (Corning Inc., Corning, NY, USA); Matrigel (BD Biosciences, San Jose, CA, USA); primary antibodies against EGFR (#4267s), phospho‐EGFR (#3777), ERK1/2 (#4695), phospho‐ERK1/2 (#4370s), Smad3 (#9523), phospho‐Smad3 (#9520; Cell Signaling Technology, Beverly, MA, USA); mouse monoclonal antibody against β‐actin (Sigma‐Aldrich); ChIPAb + TMSp1 (#17‐601; Millipore, Billerica, MA, USA); and secondary antibodies and rabbit IgG (Santa Cruz Biotechnology, Santa Cruz, CA, USA).

### 2.2 Immunohistochemical staining

Sixty‐seven invasive breast cancer tissue specimens were obtained from the Tumor Bank Facility of Tianjin Medical University Cancer Institute and Hospital in accordance with the approval of the Ethics Committee of Tianjin Medical University. The median follow‐up time was 52 months (2–75 months). Immunohistochemical (IHC) staining was performed as described previously and in Appendix S1 (Wang *et al*., 2015).

### 2.3 Cell culture and drug treatment

Human breast cancer cell lines MDA‐MB‐231 and T47D were obtained from American Type Culture Collection, where they were characterized by DNA profiling. The cells were cultured in RPMI‐1640 medium containing 10% FBS in a humidified atmosphere at 37 °C and 5% CO_2_. For TGF‐β treatment, the cells were serum‐starved for 12 h and stimulated with 5 ng·mL^−1^ TGF‐β for 12, 24, and 48 h. Afterward, the cells were harvested and further analyzed. For drug treatment, the cells were pretreated with or without MAPK pathway inhibitor (PD98059, 20 μm), Sp1 inhibitor (MTM, 40 nm), or TGF‐β receptor inhibitor (SB431542, 20 μm) overnight and exposed to TGF‐β for 24 or 48 h. The cells were harvested and examined through quantitative PCR and western blot.

### 2.4 Western blot analysis

Western blot analysis was performed as described previously and in Appendix S1 (Zhang *et al*., 2014).

### 2.5 siRNA transfection

Small interference RNA (siRNA) targeting human EGFR, Sp1, and Smad3 and negative control siRNA (Scr) were purchased from Invitrogen (Life technologies, Carlsbad, CA, USA). siRNA were transfected using a Lipofectamine RNAmix reagent in accordance with the manufacturer's instructions. After 48 h of transfection, the cells were harvested for further experiments.

### 2.6 RNA extraction and quantitative reverse transcription PCR

Total RNA was extracted with Trizol reagent, and 1 μg of RNA was reverse‐transcribed into cDNA using an M‐MLV RT kit in accordance with the manufacturer's instructions. Quantitative RT‐PCR (qRT‐PCR) analysis was performed with a SYBR Premix Ex *Taq* kit in accordance with the manufacturer's protocol. The expression of the indicated mRNA levels was quantified through 2^−ΔΔCt^ method. β‐Actin was utilized as an internal control gene to normalize gene expression. Primers used in this study are listed in Table 1.

**Table 1:** Primers and the RT‐PCR production length

Name	Primer	Sequence	Length (bp)
EGFR	Upper	5′ ATGCTCTACAACCCCACCAC 3′	193
	Lower	5′ GCCCTTCGCACTTCTTACAC 3′	
Sp1	Upper	5′ ACACGTTCGGATGAGCTACAG 3′	144
	Lower	5′ TGGGCCTCCCTTCTTATTCTG 3′	
β‐Actin	Upper	5′ CAGAGCAAGAGAGGCATCC 3′	217
	Lower	5′ CTGGGGTGTTGAAGGTCTC 3′	
TGF‐α	Upper	5′ CTTGGAGAACAGCACGTCCC 3′	133
	Lower	5′ CTCCTGCACCAAAAACCTGC 3′	
EGF	Upper	5′ TGCCCTCAACCTTGGTTTGT 3′	107
	Lower	5′ GGTTGCATTGACCCATCTGC 3′	

### 2.7 Cell migration and invasion assays

Cell migration and invasion assays were conducted as described previously and in Appendix S1 (Han *et al*., 2012; Zhang *et al*., 2015).

### 2.8 ChIP

ChIP assays were performed using a ChIP kit (Millipore), and the details are provided in Appendix S1. Primers used in ChIP assay are listed in Table 2.

**Table 2:** Primers used in ChIP assay

Name	Primer	Sequence	Length (bp)
Sp1 (‐234/‐212)	Upper	5′ CTGGGAACGCCCCTCTC 3′	149
	Lower	5′ AACCAGCAGCGGGGAC 3′	
Sp1 (‐83/‐73)/(‐64/‐52)	Upper	5′ CCCGCTGCTGGTTCTCCTCCCTC 3′	141
	Lower	5′ GGCTGCCCGGACGTCTAGCTC 3′	
Smad3 (‐1240/‐1231)	Upper	5′ TGACTTCAACGCACAGTGGC 3′	200
	Lower	5′ CTTTCCTCCTCATCCAGCAA 3′	
Smad3 (‐416/‐407)	Upper	5′ CCAGCCTCTGATCCCCGAGA 3′	148
	Lower	5′ TACAAAGCAAACTTGTACCAGC 3′	
Positive control (anti‐RNA polymerase II)	Upper	5′ TACTAGCGGTTTTACGGGCG 3′	166
	Lower	5′ TCGAACAGGAGGAGCAGAGAGCGA 3′	
Negative control	Upper	5′ AGGCTGTGAGCTAGAGCCCTAACTG 3′	289
	Lower	5′ AGCACAATACTGGGATGGATTCCAGGGAAC 3′	

### 2.9 Vector construction and dual‐luciferase reporter assay

The EGFR promoter region containing Smad3‐ and/or Sp1‐binding sites was amplified by PCR and cloned into a pGL3‐basic luciferase reporter vector in KpnI and XhoI sites. The first vector containing Smad3‐ and Sp1‐binding sites, which span from −1347 to +223 relative to the transcription initiation site, was designated as pGL3‐E1. The second vector comprising the Sp1‐binding sites (−268 to +223) was assigned as pGL3‐E2. The third vector composed of Smad3‐binding sites (−1347 to −273 and −14 to +223) was labeled as pGL3‐E3. To investigate whether Sp1 or Smad3 regulates EGFR promoter activity, we co‐transfected the control and Sp1‐ or Smad3‐silencing cells with EGFR promoters and pRL‐TK vector, which was used as an internal control, using Lipofectamine 2000. After 24 h of transfection, the cells were serum‐starved for 12 h and stimulated with 5 ng·mL^−1^ TGF‐β for 24 h. The cells were lysed, and luciferase activity was determined with a dual‐luciferase reporter assay system in accordance with the manufacturer's protocol. The EGFR promoter activity was quantified in terms of the relative value of Luc/Ren. The experiments were performed at least thrice. Primers used in this study are listed in Table 3.

**Table 3:** Primers used in dual‐luciferase reporter assay

Name	Primer	Sequence	Length (bp)
pGL3‐E1	Upper	5′ ATGACTTCAACGCACAGTGGCT 3′	1570
	Lower	5′ CCGGCTCTCCCGATCAATACT 3′	
pGL3‐E2	Upper	5′ GCCCCTCTCGGAAATTAACTCCT 3′	491
	Lower	5′ CCGGCTCTCCCGATCAATACT 3′	
pGL3‐E3	Primer 1 upper	5′ ATGACTTCAACGCACAGTGGCT 3′	1311
	Primer 1 lower	5 ′ GGCAGTGCTGGACGTCCGGGCAGCCCCCGG 3 ′	
	Primer 2 upper	5 ′ GGCAGTGCTGGACGTCCGGGCAGCCCCCGG 3 ′	
	Primer 2 lower	5 ′ CCGGCTCTCCCGATCAATACT3 ′	

### 2.10 Statistical analysis

Data were quantitatively analyzed with Student's *t*‐test or one‐way ANOVA in graphpad Prism 5.02 (GraphPad Software, San Diego CA, USA). Results were presented as mean ± SD of three independent experiments, and all of the experiments were repeated thrice. Two‐sided *P* < 0.05 was considered statistically significant. spss 17.0 software (SPSS Inc, Chicago, IL, USA) was used for clinical analysis. Chi‐square test and nonparametric Spearman's rank test were conducted to examine the relative expression and correlation between EGFR and TGF‐β. Survival plots were drawn using Kaplan–Meier method. Log‐rank test was carried out to compare the survival difference between groups.

## 3 Results

### 3.1 Increased TGF‐β expression is positively correlated with EGFR in breast tumor tissues

**Figure 1:**
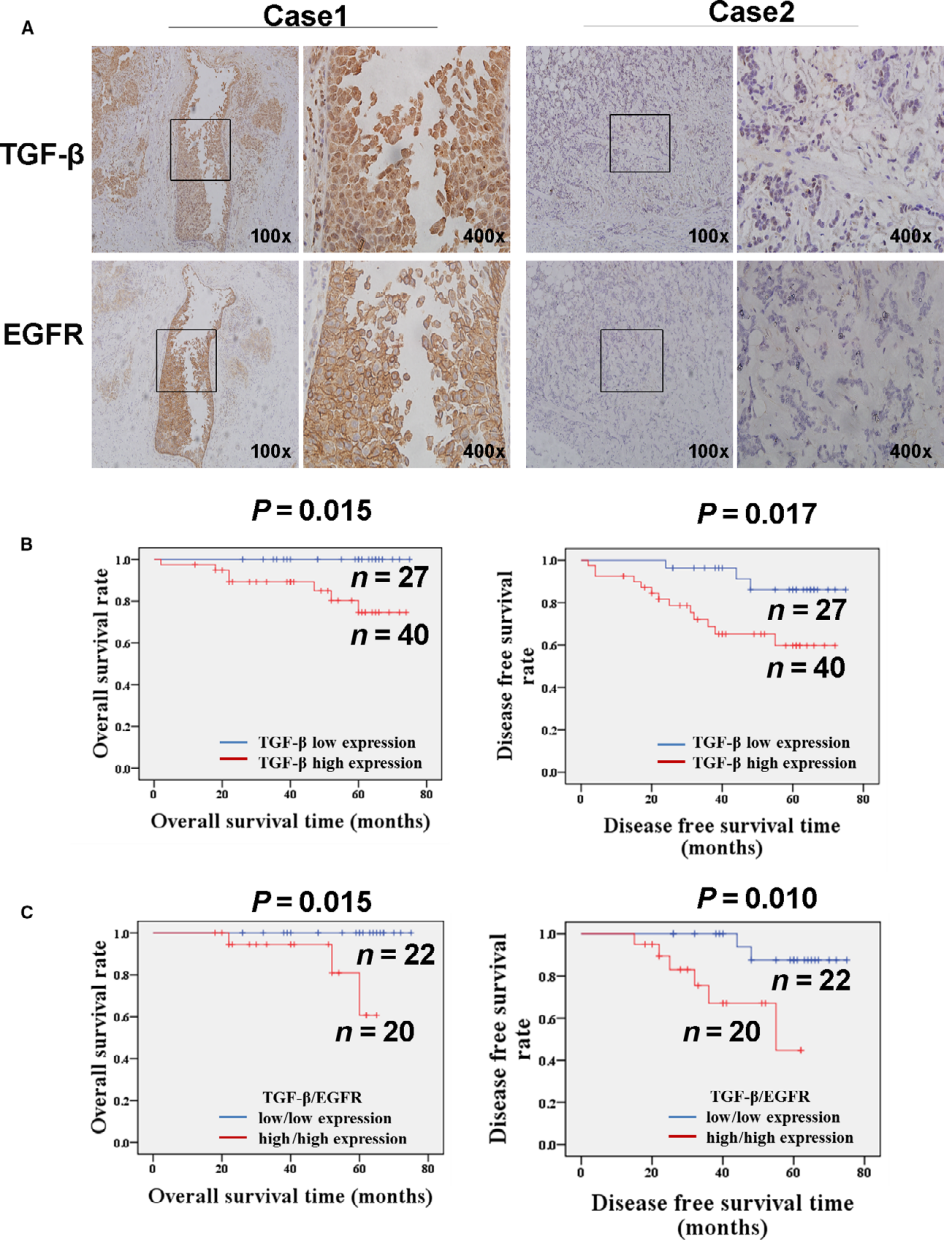
Elevated expression of TGF‐β is positively correlated with EGFR in breast cancer tissues. (A) The increased staining intensity of TGF‐β is positively correlated with EGFR elevation in invasive ductal breast cancer (IDC). The expression of TGF‐β and EGFR in breast cancer tissue was detected by IHC staining method. Case 1 was synchronously positive expression of TGF‐β and EGFR. Case 2 showed negative expression of both TGF‐β and EGFR. (B) The Kaplan–Meier method was used for survival analysis. The OS and DFS rates in patients with elevated expression of TGF‐β were significantly worse than those in patients with low expression of TGF‐β (OS,* P *=* *0.015; DFS,* P *=* *0.017). The prognosis of the patients with synchronously highly expressed TGF‐β and EGFR was much worse than that of the patients with synchronously poorly expressed TGF‐β and EGFR (OS *P *=* *0.015; DFS *P *= 0.010).

Transforming growth factor‐β functions as a pro‐metastatic factor in advanced breast cancer. High EGFR expression is correlated with the aggressiveness of cancer and the poor prognosis of patients with breast cancer. However, whether an association exists between TGF‐β and EGFR remains unknown. In this study, the relationship between TGF‐β and EGFR expression in 67 breast cancer tissues was investigated through immunohistochemistry. As shown in Fig. 1A and Table 4, TGF‐β was positively expressed in 59.7% of breast cancer tissues (40/67), EGFR expression was upregulated in 37.3% of breast cancer tissues (25/67), and EGFR and TGF‐β were co‐expressed in 29.9% of breast cancer tissues (20/67). Further statistical analysis indicated that the increased staining intensity of TGF‐β was significantly and positively correlated with EGFR elevation (Spearman's *r *=* *0.319; *P *=* *0.008). Consistent with those in a previous study, the patients with upregulated TGF‐β expression in our study showed a significantly poor overall survival (*P *=* *0.015) and disease‐free survival (*P *= 0.017; Fig. 1B). Survival analysis revealed that the prognosis of the patients with highly expressed TGF‐β and EGFR was much worse than that of the patients with poorly expressed TGF‐β and EGFR (Fig. 1C, OS *P *=* *0.015; DFS *P *=* *0.010). Our results revealed that TGF‐β expression is positively correlated with EGFR expression in breast cancer tissues, and increased TGF‐β and EGFR levels are associated with the poor prognosis of patients with breast cancer.

**Table 4:** Correlation between TGF‐β and EGFR expression

	TGF‐β expression (%)	
	Low	High
EGFR		
Low	22 (52.4)	20 (47.6)
High	5 (20)	20 (80)

### 3.2 TGF‐β promotes the increase in EGFR expression and the activation of MAPK and Smad3 signaling pathways

**Figure 2:**
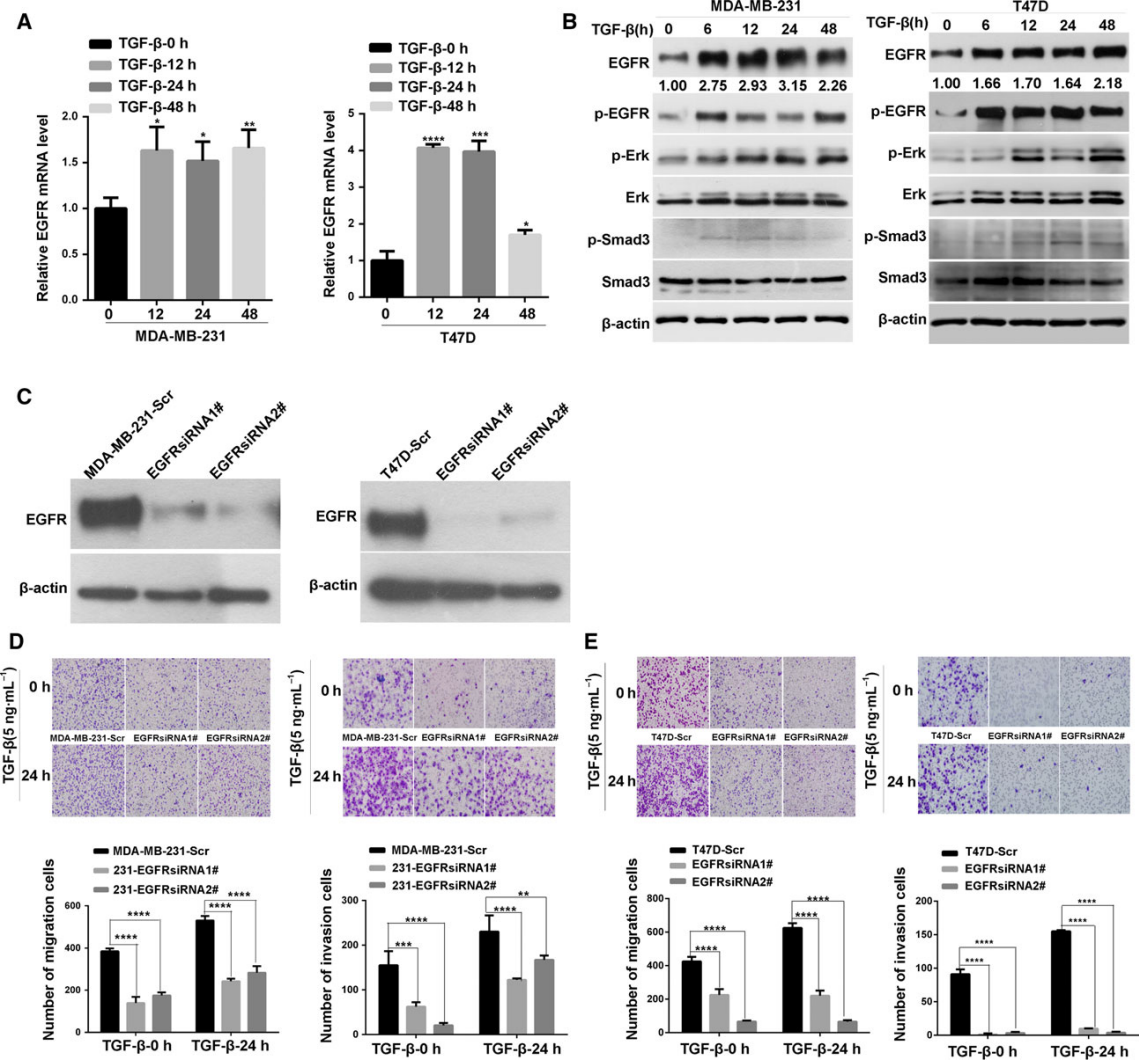
TGF‐β promotes the increase in EGFR expression and the activation of MAPK and Smad3 signaling pathways. (A) Quantitative PCR analysis of the expression level of EGFR mRNA in two breast cancer cells after TGF‐β treatment for indicated times. Statistical analysis was performed using one‐way ANOVA. *P < 0.05; **P < 0.01; ***P < 0.001; ****P < 0.0001. (B) Western blot analysis of the expression level of total and phosphorylated EGFR, total and phosphorylated Erk1/2 proteins, and total and phosphorylated Smad3 in cell lysates from two breast cancer cells treated with TGF‐β for indicated times; β‐actin was used as a loading control. (C) Western blot analysis of the expression of EGFR in cell lysates from MDA‐MB‐231 or T47D cells transfected with control and EGFR‐specific siRNA. A scrambled (Scr) sequence was used as a negative control for siRNA transfection. (D,E) Knockdown of EGFR decreases the invasion ability induced by TGF‐β. The assays were performed in triplicate and repeated three times (×200 magnification). Statistical analysis was performed by two‐way ANOVA. *P < 0.05.

To investigate whether a functional linkage exists between TGF‐β and EGFR signaling pathways, we treated two breast cancer cells with TGF‐β for 48 h and examined the mRNA and protein expression levels of EGFR. As shown in Fig. 2A,B, the mRNA expression level of EGFR in two breast cancer cells was significantly increased after the TGF‐β treatment was administered. Likewise, TGF‐β also induced a significant increase in EGFR protein level. The phosphorylation of EGFR, ERK1/2 and Smad3 was also upregulated in the TGF‐β‐treated cells. These results suggested that TGF‐β induced the transactivation of the EGFR signaling pathway at least through the upregulation of the EGFR in breast cancer cells. In addition, the migration and invasion abilities of the TGF‐β‐treated breast cancer cells were significantly enhanced compared with those of the untreated cells (Fig. S1A,B). Therefore, TGF‐β not only transactivates the EGFR signaling but also promotes the migration and invasion abilities of breast cancer cells.

### 3.3 EGFR is essential for the TGF‐β‐induced enhancement of migration and invasion abilities of breast cancer cells

To investigate whether EGFR plays a vital role in TGF‐β‐induced enhancement of the migration and invasion abilities of breast cancer cells, we knocked down the expression of EGFR in MDA‐MB‐231 and T47D cells using two different siRNA (Fig. 2C). Then, the cell migration and invasion abilities were quantified through a transwell‐based assay. Compared with that of the untreated control groups, the EGFR knockdown significantly inhibited the TGF‐β‐induced enhancement of the migration and invasion abilities of two breast cancer cells (Fig. 2D,E). In addition, an EGFR inhibitor erlotinib was used to investigate the potential effect of EGFR inhibition on TGF‐β‐induced cell migration and invasion abilities. As shown in Fig. S3A,B,C, erlotinib treatment significantly inhibited the TGF‐β‐induced enhancement of the migration and invasion abilities of two breast cancer cells. These results implied that EGFR is essential for the TGF‐β‐promoted migration and invasion abilities of breast cancer cells.

### 3.4 Sp1 and Smad3 bind to the EGFR promoter region and regulate the transcriptional activity of EGFR

**Figure 3:**
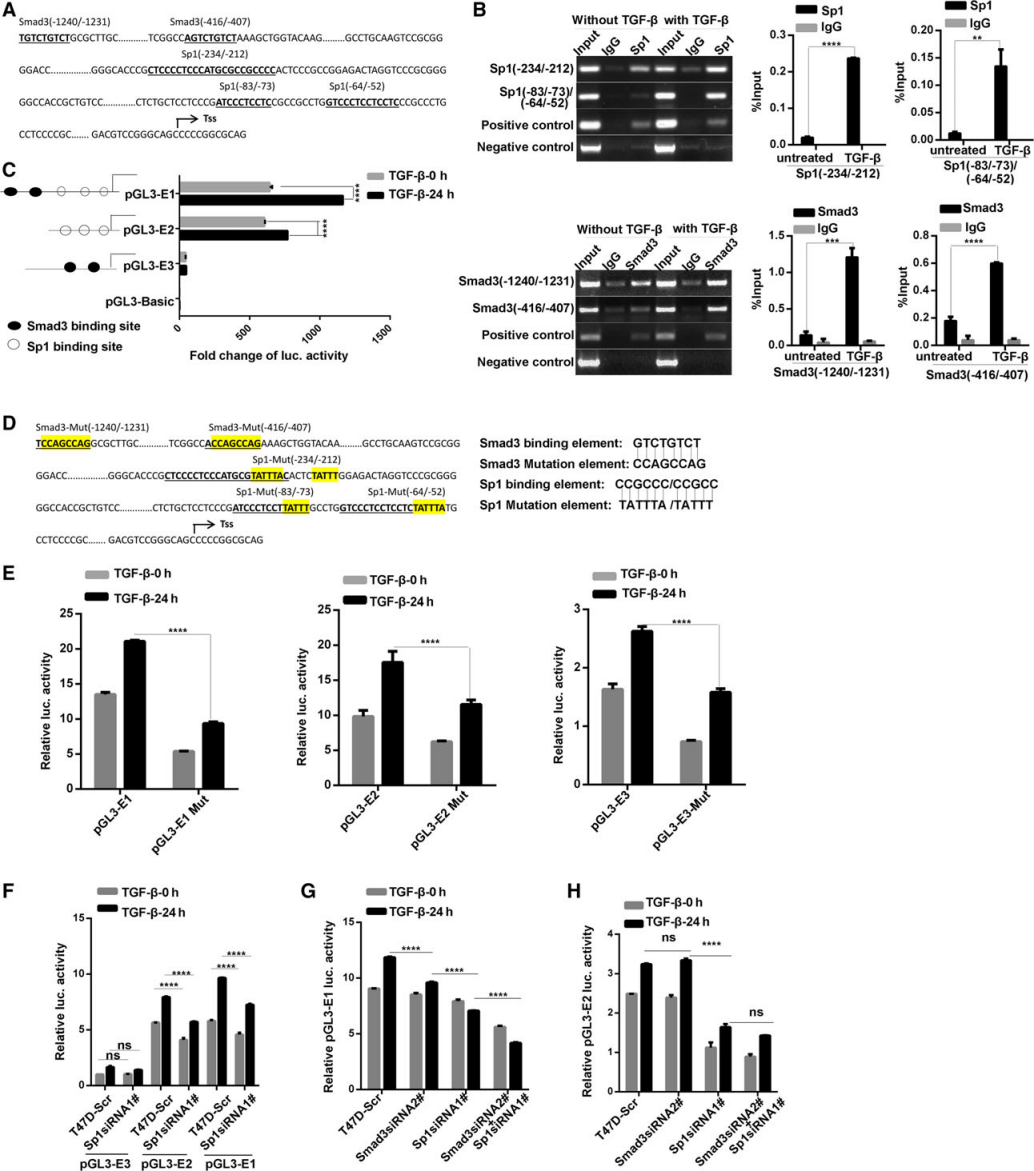
Sp1 and Smad3 bind to the EGFR promoter region and regulate the transcriptional activity of EGFR. (A) Schematic diagram depicting the regulatory sequences of the EGFR promoter region. Three putative binding sites for the transcription factor Sp1 span from −234 to −212, −83 to −73, and −64 to −52 relative to the transcription start site (TSS). The regions between −1240 to −1231 and −416 to −407 were sequences containing putative Smad3‐binding sites. (B) Chromatin immunoprecipitation assay showed that Sp1 and Smad3 specifically immunoprecipitated with EGFR promoter region. T47D cells were pretreated with or without TGF‐β for 6 h, and then, the cell lysates were subjected to chromatin immunoprecipitation using anti‐Sp1 and anti‐Smad3 antibodies. Anti‐RNA polymerase II antibody was used as a positive control. The sequences at −234 to −212, −83 to −73, and −64 to −52 with three Sp1 putative binding sites were specifically immunoprecipitated with anti‐Sp1 antibody. The binding capacity of Sp1 to the EGFR promoter was notably higher in the TGF‐β‐treated group than in the control group. The sequences at −1240 to −1231 and −416 to −407 with two Smad3 putative binding sites were specifically immunoprecipitated with anti‐Smad3 antibody, and TGF‐β treatment significantly enhanced the binding capacity of Smad3 to the EGFR promoter. Right panel, real‐time PCR analysis of the ChIP fragments. Results were analyzed by a percentage of input DNA from TGF‐β‐treated group and the control group. Statistical analysis was carried out by two‐way ANOVA (**P < 0.01, ***P < 0.001). (C) Dual‐luciferase reporter assay showed that the luciferase activity from vectors containing Sp1‐binding sites was stronger than that of other plasmids. TGF‐β treatment can also induce an apparent increase in the luciferase activation from vectors containing Sp1 sequences. Statistical analysis was carried out by two‐way ANOVA (****P < 0.0001). (D) Schematic diagram depicting the regulatory mutation sequences of the EGFR promoter region. The Sp1‐ and Smad3‐binding elements in the pGL3‐E1, E2, and E3 vectors were mutated by PCR‐mediated site‐directed mutagenesis. (E) The luciferase activities of pGL3‐E1/E2/E3 mutants were significantly weaker than those of their corresponding wild‐type plasmids. Statistical analysis was carried out by two‐way ANOVA (****P < 0.0001) (F) Knockdown of Sp1 inhibits the basal and TGF‐β‐induced luciferase activity in pGL3‐E1‐ and pGL3‐E2‐transfected cells, which contain the three putative Sp1‐binding sites. (G,H) Luciferase reporter assay showed that both Sp1 and Smad3 regulate TGF‐β‐induced EGFR transcriptional activation. Downregulation of Smad3 moderately inhibited TGF‐β‐induced luciferase activity in pGL3‐E1‐transfected cells but not in pGL3‐E2‐transfected cells, whereas double knockdown of Sp1 and Smad3 showed the strongest inhibition of luciferase activity among the other cases. All the data were presented as mean ± SD, and the experiments were repeated three times. Statistical analysis was carried out by two‐way ANOVA (****P < 0.0001).

To investigate the mechanism by which TGF‐β upregulates EGFR expression in breast cancer cells, we analyzed the EGFR promoter region using online bioinformatics tools. In Fig. 3A, three putative binding sites for the transcription factor Sp1 were at −234 to −212, −83 to −73, and −64 to −52 relative to the transcription initiation site. The regions between −1240 to −1231 and −416 to −407 were putative Smad3‐binding sites. Next, ChIP assay was performed to validate whether Sp1 or Smad3 directly binds to these predicted sites. In Fig. 3B, the sequence with Sp1‐binding sites or Smad3‐binding sites was specifically immunoprecipitated with anti‐Sp1 antibody or anti‐Smad3 antibody. In addition, quantitative PCR analysis of the ChIP fragments also showed that the binding capacity of Sp1 or Smad3 to the EGFR promoter was notably higher in the TGF‐β‐treated group than in the control group (Fig. 3B).

To determine whether Sp1 or Smad3 binds to the EGFR promoter to active this promoter after TGF‐β treatment, a panel of firefly luciferase reporter plasmids with the EGFR promoter region were examined through transient transfection assays. As shown in Fig. 3C, the luciferase activation of pGL3‐E1 (containing Smad3‐ and Sp1‐binding sites) and pGL3‐E2 (containing Sp1‐binding sites) was significantly higher than that of pGL3‐E3 (containing Smad3‐binding sites). The luciferase activity of pGL3‐E1 was stronger than that of other plasmids, and pGL3‐E3 exhibited a weak activity of the EGFR promoter. TGF‐β treatment can induce an apparent increase in the luciferase activation of pGL3‐E1 and pGL3‐E2. These findings suggested that the role of Sp1 might be more essential than that of Smad3 in the TGF‐β‐induced transcriptional activation of the EGFR promoter. Moreover, the Sp1‐ and Smad3‐binding elements in the pGL3‐E1, E2, and E3 vectors were mutated by PCR‐mediated site‐directed mutagenesis (Fig. 3D). As shown in Fig. 3E, the luciferase activities of pGL3‐E1/E2/E3 mutants were significantly weaker than those of their corresponding wild‐type plasmids. These results further suggested that Sp1 and Smad3 are involved in the TGF‐β‐induced transcriptional activation of the EGFR promoter.

To determine whether Sp1 is necessary for TGF‐β‐induced EGFR transcriptional activation, we performed a dual‐luciferase reporter assay on Sp1‐silencing T47D cells (Fig. 3F). The knockdown of Sp1 significantly decreased the basal and TGF‐β‐induced luciferase activity in pGL3‐E1‐ and pGL3‐E2‐transfected cells. By comparison, the silencing of Sp1 did not significantly affect the EGFR promoter activation in pGL‐E3‐transfected cells. To investigate the potential effect of Smad3 on TGF‐β‐induced EGFR promoter activation, we further conducted luciferase reporter assays using Smad3‐knockdown T47D cells. As shown in Fig. 3G,H, the silencing of Smad3 moderately inhibited TGF‐β‐induced luciferase activity in pGL3‐E1‐transfected cells but not in pGL3‐E2‐transfected cells. The inhibition of the promoter activation in Sp1‐silenced cells was stronger than that in Smad3‐knockdown cells. The double knockdown of Sp1 and Smad3 in pGL3‐E1‐transfected cells showed the strongest inhibition of luciferase activity among the other cases. By contrast, the silencing of Sp1 and Smad3 did not differ from the single knockdown of Sp1 in pGL3‐E2‐transfected cells in terms of luciferase activity. These results suggested that Sp1 and Smad3 bind to the EGFR promoter, and the effect of Sp1 may be stronger than that of Smad3 on TGF‐β‐induced EGFR transcriptional activation.

### 3.5 Inhibition of Sp1 weakens TGF‐β‐induced upregulation of EGFR in breast cancer cells

**Figure 4:**
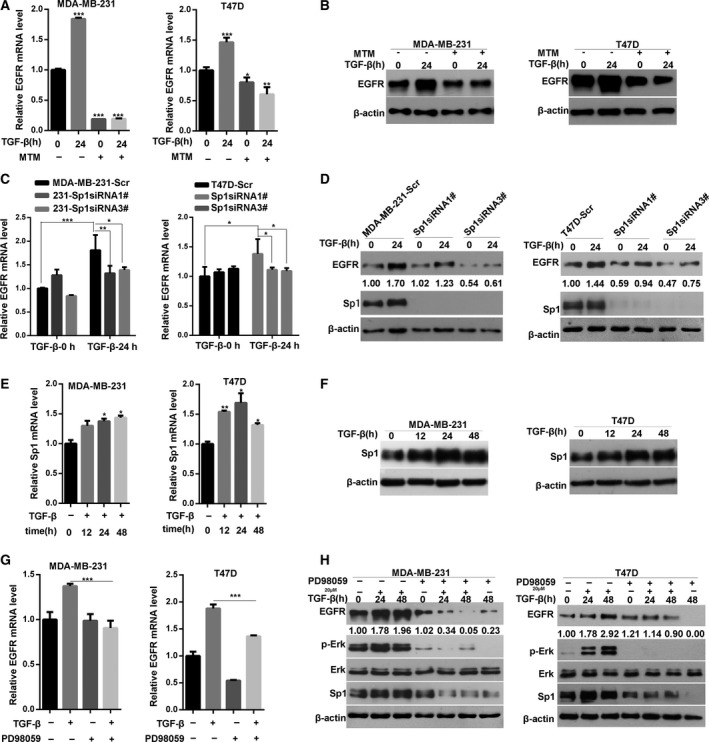
Erk/Sp1 signaling pathway is involved in TGF‐β‐induced EGFR upregulation. (A) Inhibition of Sp1 by MTM weakens TGF‐β‐induced upregulation of EGFR mRNA in breast cancer cells. Two breast cancer cells were pretreated with Sp1‐nonspecific inhibitor MTM overnight and then treated with TGF‐β for 24 h. EGFR mRNA levels were determined by quantitative PCR analysis. Statistical analysis was performed by one‐way ANOVA (*P < 0.05). (B) MTM significantly inhibited the TGF‐β‐induced increase in the protein expression levels of EGFR. (C) Knockdown of Sp1 inhibits TGF‐β‐induced upregulation of EGFR mRNA. Two breast cancer cells were transfected with control or Sp1‐specific siRNA, then treated with TGF‐β for 24 h; the expression level of EGFR mRNA was determined by quantitative PCR analysis. Statistical analysis was carried out by two‐way ANOVA (*P < 0.05; **P < 0.01; ***P < 0.001). (D) Western blot analysis of the effect of Sp1 knockdown on TGF‐β‐induced upregulation of EGFR protein level. (E) Quantitative PCR analysis of the expression level of Sp1 mRNA in two breast cancer cells treated with TGF‐β for indicated times. Statistical analysis was carried out by one‐way ANOVA (*P < 0.05; **P < 0.01). (F) Western blot analysis of the expression level of Sp1 protein in two breast cancer cells treated with TGF‐β for the indicated times. (G) Inhibition of Erk1/2 signaling by PD98059 weakens TGF‐β‐induced upregulation of EGFR mRNA in two breast cancer cells. (H) Western blot analysis of the expression level of EGFR, Sp1, total and phosphorylated Erk1/2 proteins in cell lysates from two breast cancer cells pretreated with PD98059 and then stimulated with TGF‐β for the indicated times. Statistical analysis was carried out by two‐way ANOVA (***P < 0.001).

To verify whether Sp1 is essential for the transcriptional activation of EGFR, we treated breast cancer cells with a Sp1 inhibitor MTM. Compared with that in the untreated control cells, MTM significantly inhibited the TGF‐β‐induced increase in the mRNA and protein expression levels of EGFR (Fig. 4A,B). To confirm the effect of Sp1 on TGF‐β‐induced EGFR expression, the Sp1 expression was significantly downregulated by two different Sp1 siRNA target sequences (Fig. 4C,D). Consequently, TGF‐β failed to upregulate the mRNA and protein expression of EGFR in Sp1‐silenced breast cancer cells (Fig. 4C,D). These data suggested that Sp1 is essential for the TGF‐β‐induced upregulation of EGFR expression.

### 3.6 ERK/Sp1 signaling pathway is involved in TGF‐β‐induced EGFR upregulation

Considering that Sp1 is critical for the TGF‐β‐induced upregulation of EGFR, we hypothesized that Sp1 expression is regulated by TGF‐β signaling. To test this possibility, we stimulate the breast cancer cells with TGF‐β and determined the Sp1 expression through RT‐PCR and western blot. In Fig. 4E,F, the mRNA and protein expression levels of Sp1 were significantly increased in the cells exposed to TGF‐β. Sp1 acts as a downstream molecule of the MAPK/ERK1/2 pathway, and TGF‐β can induce ERK signaling activation (Qureshi *et al*., 2005). To clarify the role of ERK1/2 in TGF‐β‐induced increase in EGFR expression, we pretreated the breast cancer cells with the ERK1/2 signaling inhibitor PD98059 and then stimulated these cells with TGF‐β. As shown in Fig. 4G,H, PD98059 inhibited the TGF‐β‐triggered phosphorylation of ERK1/2. As expected, the mRNA expression of EGFR was significantly inhibited (Fig. 4G). The inhibition of ERK1/2 signaling also significantly suppressed the TGF‐β‐induced Sp1 expression and EGFR upregulation (Fig. 4H). These results demonstrated that the ERK/Sp1 signaling pathway is implicated in the TGF‐β‐induced EGFR upregulation.

### 3.7 Knockdown of Smad3 significantly affects TGF‐β‐induced increase in EGFR expression

**Figure 5:**
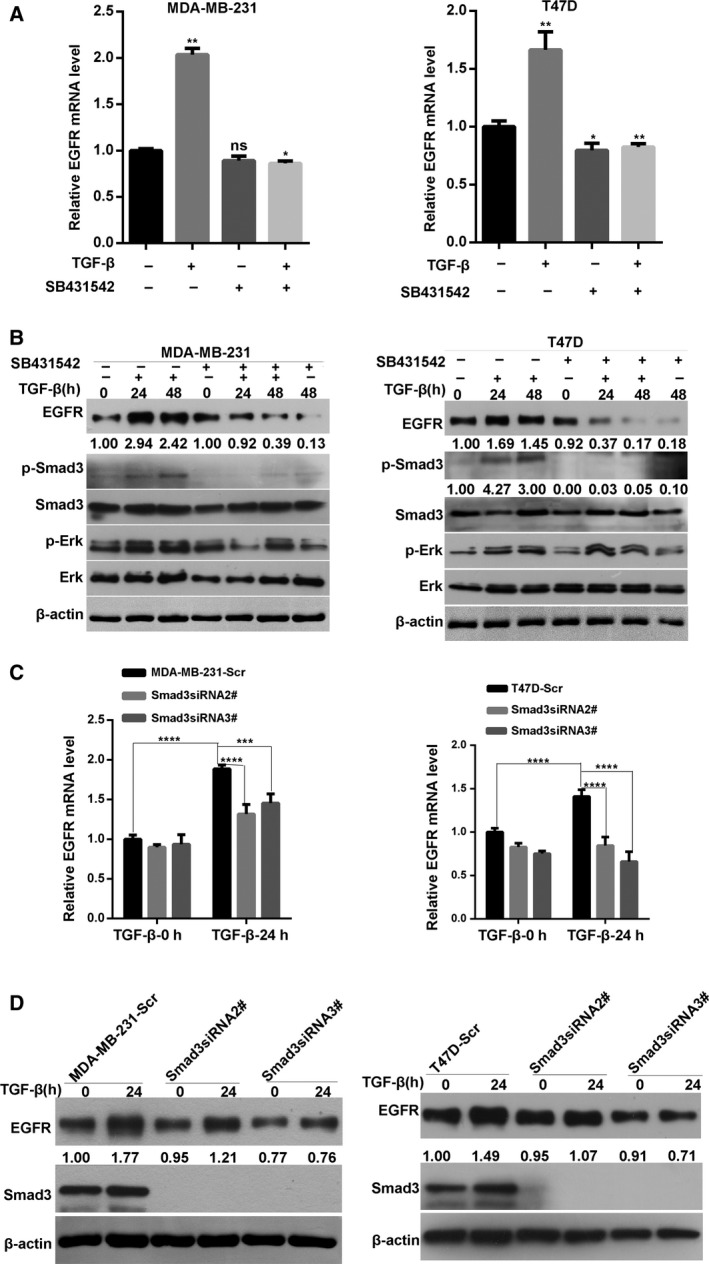
Inhibition of Smad3 pathway significantly affects TGF‐β‐induced increase in EGFR expression. (A) Quantitative PCR analysis of the expression level of EGFR mRNA in two breast cancer cells pretreated with SB431542 and then stimulated with TGF‐β for 24 h. Statistical analysis was performed by one‐way ANOVA (*P < 0.05; **P < 0.01 ns: no significant) (B) Western blot analysis of EGFR, total and phosphorylated Smad3, total and phosphorylated ERK protein expression in cell lysates from two breast cancer cells pretreated with SB431542 and then stimulated with TGF‐β for the indicated times. (C) Knockdown of Smad3 weakens TGF‐β‐induced upregulation of EGFR mRNA in breast cancer cells. Statistical analysis was carried out by two‐way ANOVA (***P < 0.001; ****P < 0.0001). (D) Western blot analysis of the effect of Smad3 knockdown on TGF‐β‐induced upregulation of EGFR protein level.

To examine the effect of the Smad3 signaling pathway on EGFR expression in breast cancer cells, the cells were pretreated with the TGF‐β receptor inhibitor SB431542 and then stimulated with TGF‐β. As shown in Fig. 5A,B, SB431542 significantly inhibited the TGF‐β‐induced Smad3 phosphorylation, indicating the inhibition of canonical TGF‐β signaling. Compared with that of the control cells, the TGF‐β‐induced upregulation of the mRNA and protein expression of EGFR was inhibited. To confirm the effect of Smad3 on TGF‐β‐induced EGFR expression, the Smad3 expression in two breast cancer cells was silenced by two different siRNA (Fig. 5C,D). As expected, the Smad3 knockdown markedly suppressed the TGF‐β‐triggered upregulation of the mRNA and protein expression of EGFR in two breast cancer cells. These findings suggested that canonical Smad3 signaling also contributes to TGF‐β‐induced increase in EGFR expression in breast cancer cells.

### 3.8 Double knockdown of Sp1 and Smad3 significantly affects the TGF‐β‐induced increase in EGFR expression

**Figure 6:**
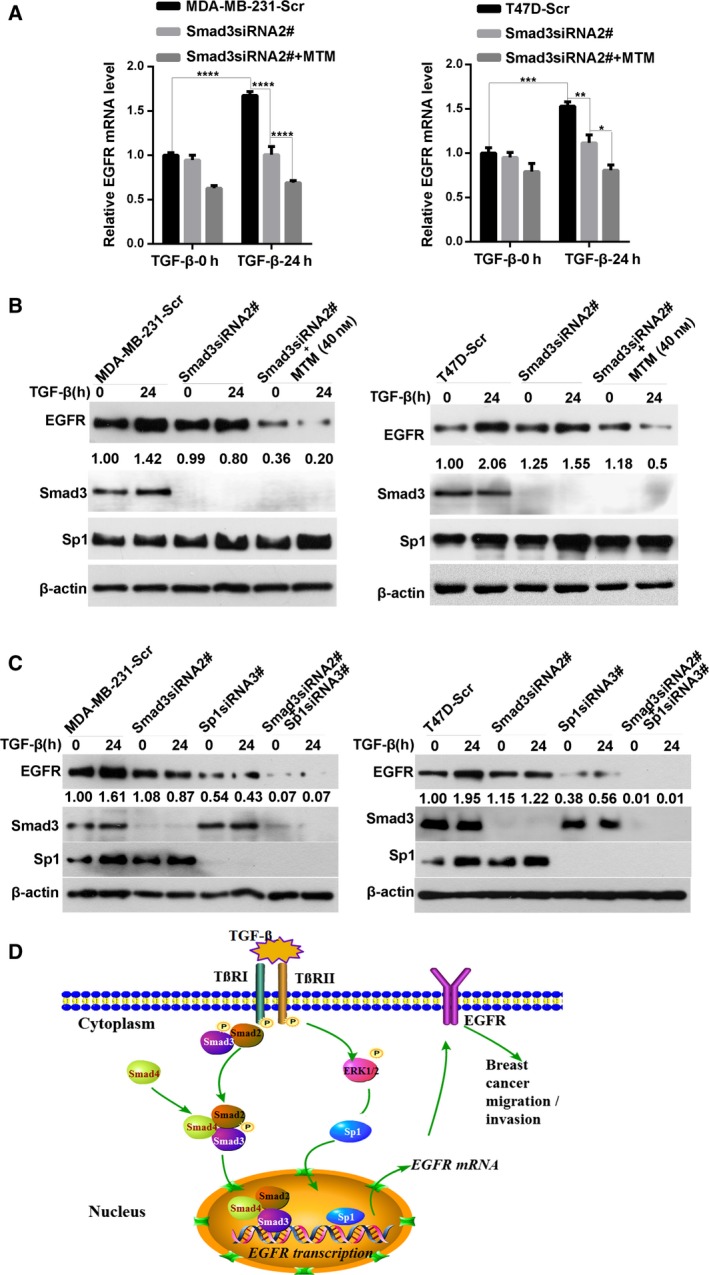
Double inhibition of Sp1 and Smad3 significantly affects the TGF‐β‐induced increase in EGFR expression. (A) Inhibition of Sp1 by MTM in Smad3‐silenced cells showed a significant decrease in the mRNA expression of EGFR. Smad3‐knockdown cells were treated with or without MTM and then stimulated with TGF‐β for 24 h. The expression of EGFR mRNA was analyzed by qRT‐PCR. Statistical analysis was carried out by two‐way ANOVA (*P < 0.05; **P < 0.01; ***P < 0.001; ****P < 0.0001). (B) Western blot analysis showed that the TGF‐β‐induced EGFR protein level in MTM‐treated Smad3‐knockdown cells was significantly lower than that of the control or Smad3‐silenced cells. (C) Double knockdown of Sp1 and Smad3 in breast cancer cells showed more strong inhibition of TGF‐β‐induced upregulation of EGFR expression than that in the single‐Sp1 and single‐Smad3 siRNA‐transfected breast cancer cells. Moreover, Sp1‐knockdown cells showed much stronger inhibitory effect on TGF‐β‐induced EGFR upregulation compared to Smad3‐knockdown cells. (D) A proposed schematic model: TGF‐β transactivates EGFR and promotes migration and invasion abilities of breast cancer cells.

To investigate whether Sp1 and Smad3 elicit a synergetic effect on TGF‐β‐induced upregulation of EGFR expression, on the basis of Smad3 knockdown, we used the Sp1 inhibitor MTM to treat breast cancer cells. Compared with that in the control cells or Smad3‐knockdown cells, the inhibition of Sp1 in Smad3‐silenced cells showed an evident decrease in the mRNA and protein expression of EGFR (Fig. 6A,B). Afterward, the EGFR protein levels in Smad3 and Sp1‐double‐silenced cells were further examined. As shown in Fig. 6C, the TGF‐β‐induced EGFR expression was more strongly inhibited by the double suppression of Smad3 and Sp1 expression than by the single‐Sp1 or single‐Smad3 siRNA‐transfected breast cancer cells. In addition, knockdown of Sp1 showed much stronger inhibitory effect on TGF‐β‐induced EGFR upregulation than that in Smad3‐knockdown cells (Fig. 6C). Moreover, quantitative analysis demonstrated that the double knockdown of Sp1 and Smad3 triggered an additive effect on EGFR expression in breast cancer cells but did not cause a synergistic effect.

### 3.9 Knockdown of Sp1 or Smad3 by siRNA prevents TGF‐β‐induced migration and invasion abilities of breast cancer cells

To further confirm the role of Sp1 and Smad3 in TGF‐β‐induced enhancement of the migration and invasion abilities of breast cancer cells, we knocked down the expression of Sp1 or Smad3 using two different siRNA sequences in two breast cancer cells (Fig. S2A,C). Then, the cell migration and invasion abilities were measured by a transwell‐based assay. As shown in Fig. S2B,D, downregulation of Sp1 or Smad3 significantly inhibited the TGF‐β‐induced enhancement of the migration and invasion abilities of two breast cancer cells.

## 4 Discussion

Transforming growth factor‐β functions as a potent proliferation inhibitor and apoptosis inducer in early stages of breast cancer, but it promotes cancer invasion and metastasis in advanced stages. This remarkable functional conversion of TGF‐β during cancer progression is known as ‘TGF‐β paradox’ (Tian and Schiemann, 2009). Several reports have suggested the cross talk between TGF‐β signaling and other pathways, including EGFR signaling during cancer progression, and this finding possibly explains why TGF‐β favors tumor progression in late stages. Although the activation of TGF‐β signaling and EGFR overexpression are often observed in invasive breast carcinomas and correlated with cancer progression (Katz *et al*., 2013; Masuda *et al*., 2012), the detailed mechanism remains unclear. In particular, the association between TGF‐β and EGFR in breast cancer is poorly described. In this study, we demonstrated that the expression of TGF‐β is positively correlated with the EGFR expression in breast cancer tissues, and the increased levels of TGF‐β and EGFR are associated with the poor prognosis of patients with cancer. The interplay between TGF‐β signaling and EGFR is also confirmed in breast cancer cell lines. TGF‐β promotes the migration and invasion abilities of breast cancer cells and increases the EGFR expression. EGFR is also essential for the TGF‐β‐induced enhancement of the migration and invasion abilities of breast cancer cells. Canonical Smad3 signaling and ERK/Sp1 pathways mediate the TGF‐β‐induced upregulation of EGFR expression. Hence, our study suggested that TGF‐β transactivates EGFR signaling by upregulating the EGFR expression in breast cancer cells.

Our findings indicated a possible cooperation between TGF‐β and EGFR in enhancing breast cancer aggravation. The synergistic effect between TGF‐β and EGFR on promoting cancer progression has also been confirmed in several cancer types (Buonato *et al*., 2015; Kang *et al*., 2012; Richter *et al*., 2011; Uttamsingh *et al*., 2008; Wendt *et al*., 2010; Xu *et al*., 2010). To support these observations, we showed that TGF‐β enhances the migration ability of breast cancer cells, increases EGFR expression, and activates the MAPK signaling pathway. These findings also implied that EGFR signaling is transactivated by TGF‐β in breast cancer cells. In EGFR‐silenced cells, TGF‐β fails to induce a more aggressive phenotype, and this result indicated that EGFR is required for the TGF‐β‐induced increase in the invasion ability of breast cancer cells. Therefore, our data suggested that TGF‐β transactivates EGFR signaling by upregulating EGFR expression and facilitates breast cancer migration and invasion.

Several studies indicated that TGF‐β induces EGFR transactivation in a highly cell‐type‐ and context‐specific manner. For instance, TGF‐β transactivates EGFR signaling in hepatocytes through EGFR ligand shedding (Moreno‐Càceres *et al*., 2014). In squamous carcinoma cells, TGF‐β induces EGFR activation by producing H_2_O_2_ without affecting EGFR protein levels (Lee *et al*., 2010). Src kinase signaling, reactive oxygen species production, and *de novo* protein synthesis are involved in TGF‐β‐induced transactivation of EGFR in HaCaT cell (Joo *et al*., 2007). Here, we showed that TGF‐β transactivates EGFR signaling through the upregulation of EGFR expression. In addition, our data also showed that the expression of EGF‐like ligands was not significantly induced by TGF‐β in two breast cancer cells (Fig. S4). This finding provided a novel mechanism by which TGF‐β transactivates EGFR signaling. In addition, multiple Sp1‐ and two Smad3‐binding sites were identified in the EGFR promoter region through bioinformatics approach. Considering that Smad3 is the downstream effector of TGF‐β signaling and Sp1 is also involved in this pathway (Greenwel *et al*., 1997; Jungert *et al*., 2007), we hypothesized that Sp1 and Smad3 may be involved in TGF‐β‐induced EGFR upregulation. Our results revealed that Sp1 and Smad3 bind to the EGFR promoter, and the binding capacity was significantly enhanced after TGF‐β treatment is administered. Moreover, the luciferase activity of pGL3‐E1 was stronger than that of other plasmids, and pGL3‐E3 exhibited a weak activity of the EGFR promoter. Thus, the role of Sp1 may be more important than that of Smad3 in TGF‐β‐induced EGFR upregulation. It is possible that the binding of Sp1 to EGFR promoter is required for Smad3‐mediated transcriptional activation of EGFR. This hypothesis was further supported by the finding that Sp1 downregulation notably decreases the TGF‐β‐induced EGFR promoter activation, whereas Smad3 silencing moderately inhibits this effect. Furthermore, double knockdown of Sp1 and Smad3 strongly blocks the luciferase activity. Thus, Sp1 and Smad3 are likely involved in TGF‐β‐induced EGFR transcription, but the effect of Sp1 may be much stronger than that of Smad3.

The inhibition of Sp1 by MTM or the silencing of Sp1 expression by siRNA can inhibit TGF‐β‐triggered EGFR upregulation. This finding further confirmed the regulatory role of Sp1 in TGF‐β‐induced EGFR expression. In this study, the Sp1 expression in breast cancer cells is increased after TGF‐β treatment is administered. It has been reported that ERK1/2 signaling acts as an upstream factor of Sp1 (Qureshi *et al*., 2005). Our data and previous findings also demonstrated that TGF‐β can induce the activation of ERK1/2 signaling. Therefore, ERK/Sp1 signaling pathway is possibly implicated in TGF‐β‐induced upregulation of EGFR expression. Consistent with this hypothesis, PD98059‐induced inhibition of ERK1/2 signaling disrupts the TGF‐β‐induced expression of Sp1. The EGFR expression is also decreased. Therefore, ERK/Sp1 signaling pathway contributes to the TGF‐β‐induced transactivation of EGFR.

Herein, the inhibition of the TGF‐β receptor using the inhibitor SB431542 can attenuate the TGF‐β‐induced EGFR upregulation. This finding indicated the involvement of canonical Smad pathway in EGFR induction. Consistently, knockdown of Smad3 in breast cancer cells also decreases the upregulated EGFR expression induced by TGF‐β treatment. These results confirmed that the canonical Smad3 signaling pathway is associated with TGF‐β‐induced EGFR upregulation. However, this finding is different from a previous report on hepatocytes, in which the canonical Smad‐mediated EGFR activation occurs through an increase in EGF ligand activity but not through the additional expression of EGFR (Kang *et al*., 2012). This inconsistency may be attributed to different cell models used in these experiments. The mechanisms by which TGF‐β transactivates EGFR may also be specific to cell type and context. These differences also implied that tumor cells can employ various mechanisms to transactivate EGFR under different conditions and thus result in cancer progression.

Sp1 was reported to collaborate with Smad3 to regulate TGF‐β‐induced CTGF transcription in myoblasts (Cordova *et al*., 2015). Sp1 also interacts with Smad and promotes MMP11 expression in colon cancer cells (Barrasa *et al*., 2012). In this study, we found that the EGFR expression was significantly lower in Smad3‐silenced breast cancer cells treated with MTM than in the cells with single‐silenced Smad3. Therefore, Sp1 possibly cooperates with Smad3 in TGF‐β‐induced EGFR expression. Consistently, the inhibition of TGF‐β‐induced EGFR expression was stronger in the cells with double knockdown of Smad3 and Sp1 expression than in the cells with single‐silenced Smad3 or Sp1. Moreover, quantitative analysis suggested that Sp1 and Smad3 caused an additive effect on EGFR expression in breast cancer cells. Collectively, we concluded that Sp1 might cooperate with Smad3 in TGF‐β‐induced EGFR expression.

## 5 Conclusions

Our study demonstrated that TGF‐β promotes the migration and invasion abilities of breast cancer cells through the upregulation of EGFR expression. Canonical Smad3 signaling and ERK/Sp1 signaling pathways are required for the TGF‐β‐induced upregulation of EGFR and the enhancement of migration and invasion abilities of breast cancer cells. Hence, our findings provided insights into a novel mechanism by which TGF‐β favors breast cancer progression.

## Author contributions

RN and FZ designed the research. YZ, JM, YF, RT, and ZW performed the research. YZ, YF, and WJ generated the data. FZ and YZ wrote the manuscript. All authors read and approved the final manuscript.

## Supporting information


**Fig. S1**. TGF‐β promotes migration and invasion ability in breast cancer cells.
**Fig. S2**. Knockdown of Sp1 or Smad3 by siRNA prevents TGF‐β‐induced migration and invasion ability of breast cancer cells.
**Fig. S3**. Inhibition of EGFR by Erlotinib significantly blocked TGF‐β‐induced the enhancement of migration and invasion in two breast cancer cells.
**Fig. S4**. The expression of EGF‐like ligands was not significantly induced by TGF‐β in two breast cancer cells.
**Appendix S1**. Supplementary materials and methods.
