# Supplementary material for: TGF‐β transactivates EGFR and facilitates breast cancer migration and invasion through canonical Smad3 and ERK/Sp1 signaling pathways
Source: Mol Oncol. 2018 Jan 24;12(3):305–21. doi: 10.1002/1878-0261.12162 (PMC5830653; doi:10.1002/1878-0261.12162)

Supplementary Materials and Methods

Immunohistochemical staining

Sixty-seven invasive breast cancer tissue specimens were obtained from the Tumor Bank Facility of Tianjin Medical University Cancer Institute and Hospital in accordance with the approval of the Ethics Committee of Tianjin Medical University. The median follow-up time was 52 months (2–75 months). Rabbit anti-human TGF-β polyclonal antibody and positive tissue control slides were procured from Fuzhou Maixin Biotech. Co., Ltd. (#RAB-0238). Rabbit anti-human EGFR antibodies were purchased from ZEGB-BIO Company (Beijing, China). TGF-β and EGFR were prepared as working concentrations. For the negative control, the primary antibodies were replaced with PBS. The expression levels of EGFR and TGF-β were evaluated by two independent pathologists. For immunohistochemical staining standards, staining intensity and positive cell percentage were combined to determine the total score. The staining intensity was scored as follows: 0, negative staining; 1, weakly positive staining; 2, moderately positive staining; and 3, strongly positive staining. The percentage of positive cells was interpreted as follows: 0, no staining observed; 1, 0%–25% positive rate; 2, 25%–50% positive rate; 3, 50%–75% positive rate; and 4, > 75% positive rate. The final score of each slide was obtained by combining the intensity and the percentage. Scores ranging from 0 to 7 and greater than 4 were defined as high expression.

Chromatin Immunoprecipitation

T47D breast cancer cells were exposed to TGF-β (5 ng/mL) or PBS for 6 h and

then cross-linked using 1% formaldehyde at 37 °C for 10 min. Afterward, 0.125 M glycine was added to each dish and incubated at room temperature for 5 min to quench unreacted formaldehyde. The cells were washed thrice with ice-cold PBS containing 1× protease inhibitor cocktail, lysed by adding 1 mL of SDS lysis buffer, sonicated, and centrifuged to remove insoluble cell debris. The supernatant was subjected to immunoprecipitation by using anti-Sp1 and anti-Smad3 antibodies. Rabbit IgG and anti-RNA polymerase II were utilized as negative and positive controls. The immune complex was enriched with protein G-agarose. The DNA–protein complexes were washed successively in low-salt immune complex wash buffer, high-salt immune complex wash buffer, LiCl immune complex wash buffer, and TE buffer. The DNA was purified using a PCR purification kit (Thermo #K0701), and the purified DNA was used as a template to amplify the promoter region of EGFR through PCR. The primers used in this procedure are described in Table 4. The PCR conditions were as follows: initial denaturation at 95 °C for 3 min; denaturation at 95 °C for 30 s; annealing at 60 °C for 30 s; extension at 72 °C for 30 s; and a final extension at 72 °C for 5 min. ChIP-PCR products were examined through electrophoresis with 2% agarose gel and stained with ethidium bromide.

Western blot

In brief, the cells were lysed with 1 × SDS lysis buffer, and total protein was quantified using Nanodrop ND-2000. The proteins were separated through SDS-PAGE and transferred onto PVDF membranes. The membranes were subsequently blocked with 5% non-fat milk for 1 h at room temperature, incubated

with primary antibodies at 4 °C overnight, and washed thrice with TBST. The membrane was then incubated with the corresponding horseradish peroxidase-linked secondary antibodies, and the expression of the indicated proteins was detected using an ECL kit.

Cell migration and invasion assay

In brief, 8 μm Transwell inserts were used for cell migration and invasion assays. For the migration assay, the cells were pretreated with or without 5 ng/mL TGF-β for 24 h. Afterward, 200 μL of cell suspension at a density of 1.5×10^5 cells/mL (T47D) or 1.0×10^5 cells/mL (MDA-MB-231) was loaded into the Transwell inserts. The lower insert was loaded with a medium containing 30 ng/mL EGF and incubated at 37 °C for 12 h to allow cell migration. Subsequently, the migrated cells were fixed, stained, and quantified using a light microscope at a magnification of 400×. For the cell invasion assay, the Transwell inserts were pre-coated with Matrigel, and the incubation time was extended to 24 h. All of the experiments were performed in triplicate and repeated thrice.

Supplementary Figure 1

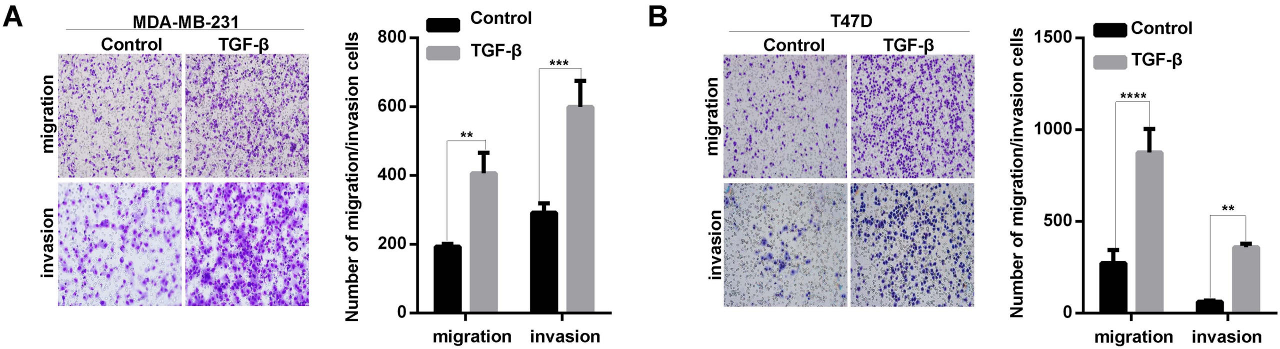

Supplementary Figure 2

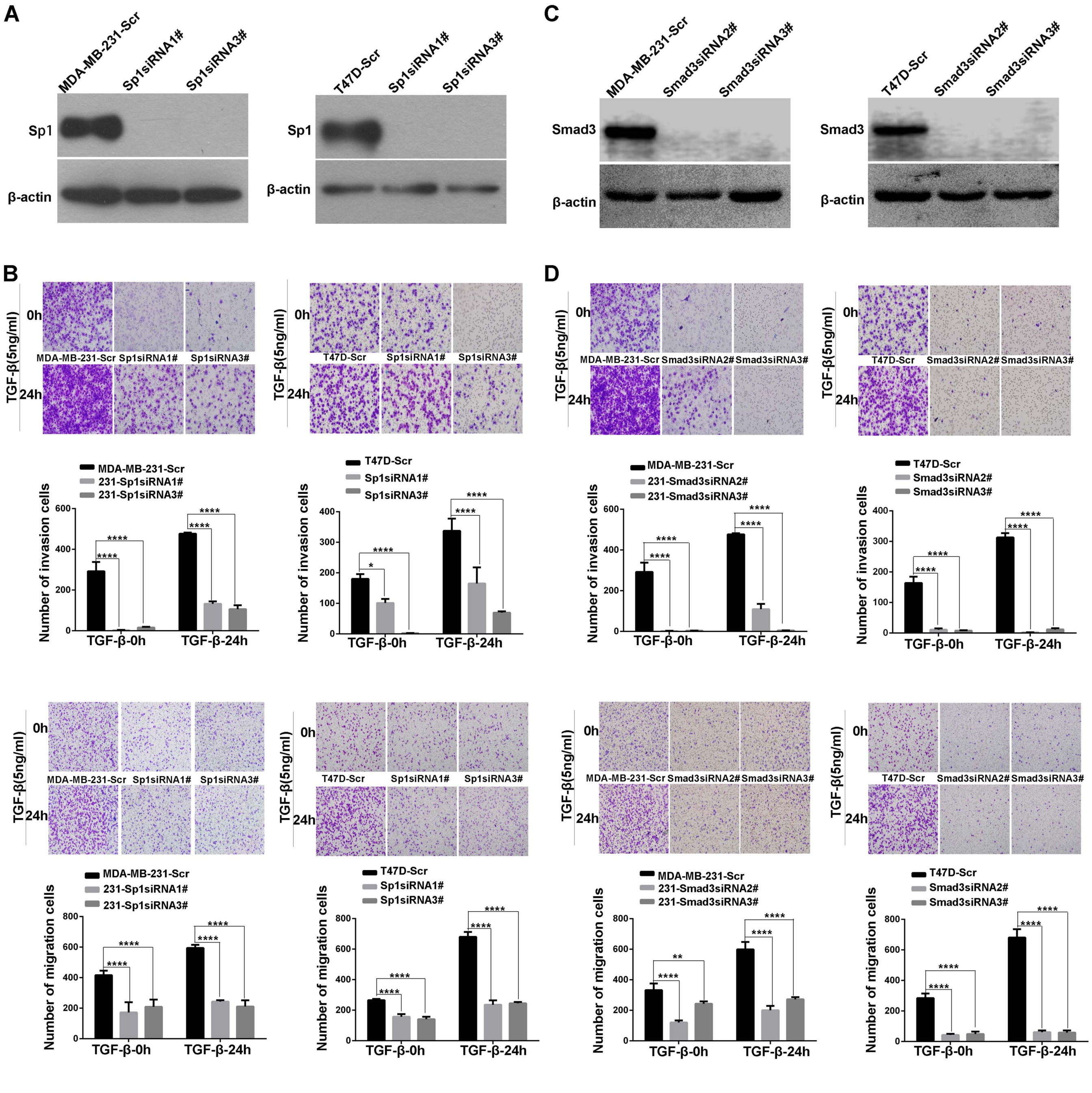

Supplementary Figure 3

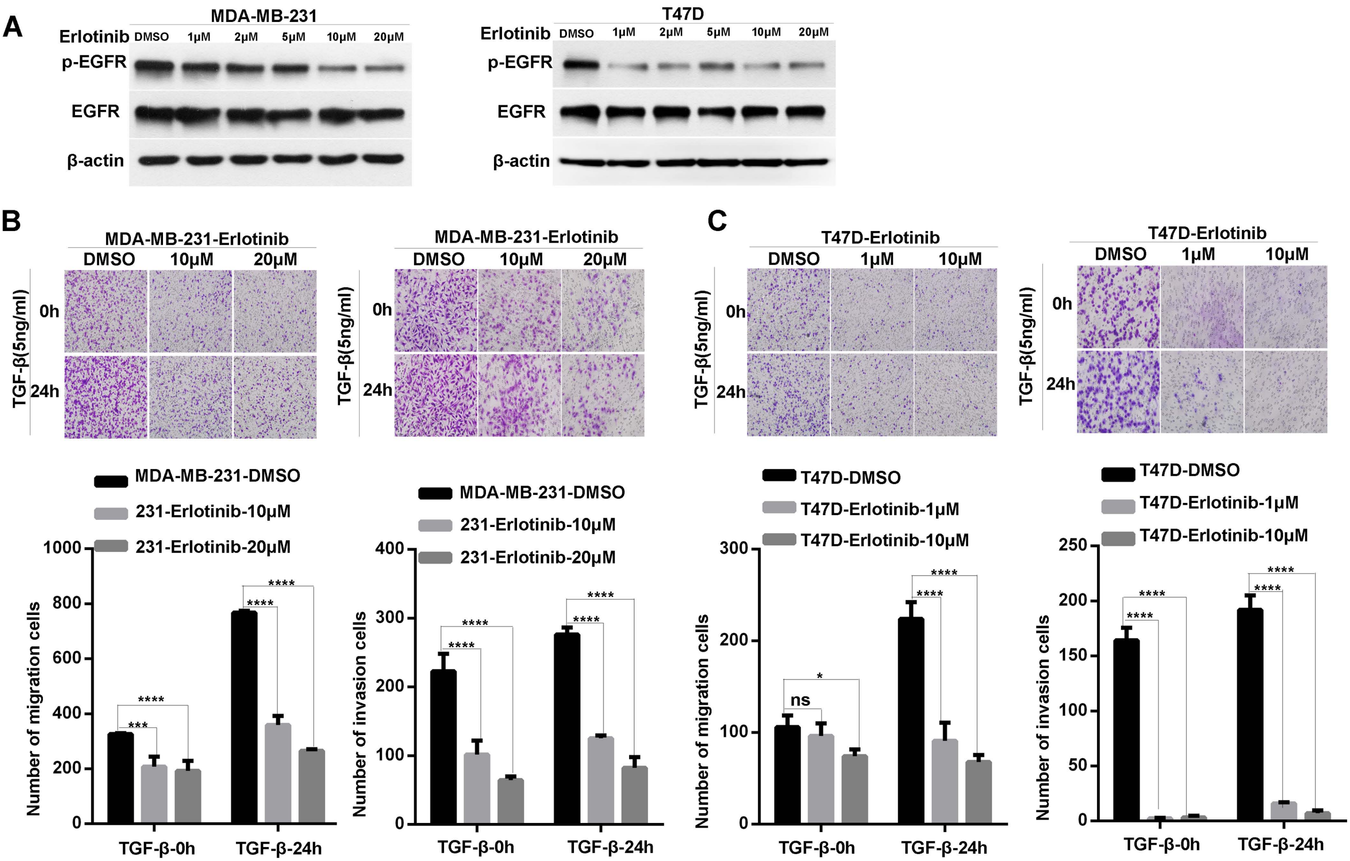

Supplementary Figure 4

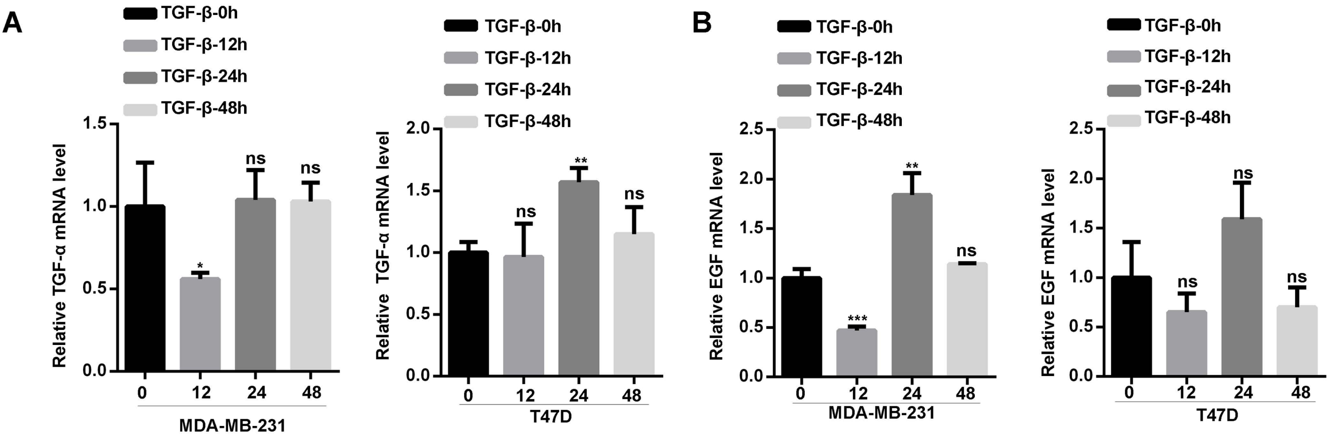

Supplement: Supplementary file 1 — Fig. S1. TGF‐β promotes migration and invasion ability in breast cancer cells. Fig. S2. Knockdown of Sp1 or Smad3 by siRNA prevents TGF‐β‐induced migration and invasion ability of breast cancer cells. Fig. S3. Inhibition of EGFR by Erlotinib significantly blocked TGF‐β‐induced the enhancement of migration and invasion in two breast cancer cells. Fig. S4. The expression of EGF‐like ligands was not significantly induced by TGF‐β in two breast cancer cells. Appendix S1. Supplementary materials and methods. [file MOL2-12-305-s001.pdf]
